# Description of the Seminiferous Epithelium Cycle Stages in the Zebra Finch *Taeniopygia guttata*

**DOI:** 10.3390/ani15233427

**Published:** 2025-11-27

**Authors:** Tatiana Bikchurina, Daria Rubtsova, Daria Odnoprienko, Pavel Borodin, Anna Torgasheva, Lyubov Malinovskaya

**Affiliations:** 1 Laboratory of Genome Structure and Function, Novosibirsk State University, 630090 Novosibirsk, Russia; t.bikchurina@g.nsu.ru; 2 Laboratory of Recombination and Segregation Analysis, Institute of Cytology and Genetics, 630090 Novosibirsk, Russia; d.rubtsova@bionet.nsc.ru (D.R.); odnoprienkodr@bionet.nsc.ru (D.O.); borodin@bionet.nsc.ru (P.B.); 3 Centre for Molecular Biodiversity Research, Leibniz Institute for the Analysis of Biodiversity Change, Museum Koenig Bonn, 53113 Bonn, Germany; a.torgasheva@leibniz-lib.de; 4 Bonn Institute for Organismic Biology—Animal Biodiversity, University of Bonn, 53121 Bonn, Germany

**Keywords:** seminiferous epithelium cycle, spermatogenesis, zebra finch, spermiogenesis, germline-restricted chromosome (GRC)

## Abstract

Spermatogenesis in passerine birds remains poorly characterized. Previous studies focused on the steps of spermiogenesis, while a detailed staging of the seminiferous epithelium cycle is still lacking for this order. In this study, we provide the first comprehensive histological characterization of spermatogenesis in a model passerine species, the zebra finch. We defined ten distinct steps of spermiogenesis based on acrosomal and nuclear morphology of spermatids in Periodic acid–Schiff-stained histological sections. Using this classification, we further defined seven stages of the seminiferous epithelium, encompassing cytological features associated with the programmed DNA elimination, which is unique to passerines. Our analysis revealed that seminiferous tubules of the zebra finch exhibit a mosaic organization typical of all birds examined so far. However, in this species, most stages were characterized by the presence of spermatids at three developmental steps, in contrast to the one to two steps typical of non-passerine birds. To facilitate future comparative studies, we developed a flowchart for determining the stages of the seminiferous epithelium cycle. This work establishes an essential histological framework for future research in reproductive biology of the zebra finch and other passerine species.

## Simple Summary

This study provides the first detailed description of the seminiferous epithelium cycle in a passerine, the zebra finch. We analyzed sequential morphological changes in developing spermatids and identified ten steps of their maturation. On this basis, we define seven stages of the seminiferous epithelium cycle and provide a concise flowchart to facilitate staging. We found that the zebra finch seminiferous epithelium cycle has a more complex organization than in non-passerine birds, as each stage contains spermatids at two to three maturation steps. Together, these resources form a reference framework for zebra finch testicular histology and will facilitate future studies in avian reproductive biology.

## 1. Introduction

In mammals and a few birds studied so far, the seminiferous epithelium cycle is divided into a series of distinct stages [1,2]. These stages of the seminiferous epithelium cycle are typically defined by the morphology of the developing spermatids. During spermiogenesis, the cells undergo sequential, easily visualized morphological changes—such as the formation of the acrosome and the elongation and condensation of the nucleus. These changes provide clear, discrete criteria for defining the “spermiogenesis steps”. Consequently, each stage of the seminiferous epithelium cycle is characterized by a specific set of spermatids at different steps [3,4,5].

Unlike the well-ordered, sequential cycle observed in mammalian testes, the avian testes display more heterogeneous and complex organization [6,7,8]. In birds, multiple stages of the seminiferous epithelium cycle are present within a single cross-section of a seminiferous tubule [9,10]. This striking difference makes particularly difficult an analysis of avian spermatogenesis, as each stage occupies only a small segment of the tubule.

To date, detailed histological descriptions of the seminiferous epithelium cycle have been conducted for only a handful of avian species, primarily domestic poultry like chickens, quails, and geese [1,3,9,11,12]. Among passerines, analyses for the house sparrow (*Passer domesticus*) [13], the carib grackle (*Quiscalus lugubris*) [14], and the masked weaver (*Ploceus velatus*) [3] are limited to the description of spermiogenesis steps. This pronounced gap in knowledge limits our understanding of avian reproductive diversity and evolution and highlights the importance of species-specific histological characterization of the seminiferous epithelium cycle in passerines. Moreover, these birds possess a unique cytogenetic feature—the germline-restricted chromosome (GRC)—which is eliminated from the male germline during spermatogenesis and needs to be considered in any comprehensive description of their testicular organization [15,16].

The zebra finch (*Taeniopygia guttata*) is an important model organism in ornithology, neurobiology, ethology, and genomics. Its well-annotated genome, ease of breeding in captivity, and well-characterized vocal learning and social behavior make it an indispensable subject for integrative biological studies [17,18]. However, a comprehensive histological characterization of the seminiferous epithelium cycle in this species is lacking.

Here, we provide the first detailed histological description of the seminiferous epithelium cycle in the zebra finch. Using classical histological techniques (paraffin sectioning, Periodic acid–Schiff (PAS) staining and fluorescence in situ hybridization (FISH)) with a GRC-specific probe, we characterized the seminiferous epithelium cycle based on the sequential steps of spermiogenesis.

## 2. Materials and Methods

### 2.1. Animals

Five mature adult zebra finch males were purchased from a pet shop in April–May 2024 and 2025. This period was selected as it corresponds to the peak of reproductive activity of the zebra finch in laboratory conditions [19]. Upon arrival, all birds were visually confirmed to be in good health, showing no signs of illness. Three birds were used for histological analysis, and two birds was used for FISH experiments. All procedures, including animal handling and euthanasia, were performed in compliance with national regulations for the use of laboratories. Birds were euthanized by an overdose of isoflurane. This study was conducted in accordance with the ARRIVE guidelines and approved by the Bioethics Committee of the Institute of Cytology and Genetics, Siberian Branch of the Russian Academy of Sciences (protocol #199, 21 November 2024).

### 2.2. Histology

Testes of four birds were immediately isolated after euthanasia and fixed in Bouin’s solution (picric acid:37% formaldehyde:glacial acetic acid, 15:5:1) for 24 h (for histological analysis) or in 10% formalin for 2–3 days (for subsequent FISH experiments). The tissues were then dehydrated through a graded ethanol series, cleared in xylene, and embedded in paraffin blocks. From these blocks, serial sections (3–4 μm thick) were obtained for both histological and FISH analyses using a rotary microtome equipped with a Section Transfer System (Microm HM355S, Thermo Fisher Scientific, Waltham, MA, USA) and mounted on slides.

The sections were deparaffinized in xylene, rehydrated through a graded ethanol series, and stained with Periodic acid–Schiff (PAS) stain using a kit from Biovitrum, Saint Petersburg, Russia (cat #HK-PS-AQ00), according to the manufacturer’s instructions. Briefly, slides were incubated in 1% periodic acid for 10 min at RT, followed by incubation in Schiff’s reagent for 20 min at RT. After washing twice in distilled water, sections were incubated in sulfurous water for 2 min at RT and counterstained with Gill’s hematoxylin for 2 min. Slides were washed in tap water for 5 min, then dehydrated in an ethanol series, cleared in xylene, and mounted in Virtogel medium (Biovitrum, Saint Petersburg, Russia, cat# 12-005).

Testes of one bird were fixed in 4% PFA in PBS overnight and washed three times in PBS for 20 min. Gelatin-embedding and cryosectioning (thickness, 18–20 μm) were performed according to a standard protocol [20].

### 2.3. FISH

The zebra finch GRC whole-chromosome DNA probe were derived from microdissected GRC muclonuclei [16]. The probe was labeled with fluorochrome-conjugated nucleotide TAMRA-5-dUTP or Flu-12-dUTP (Biosan, Novosibirsk, Russia, cat# N-312-1000) using the GenomePlex Whole Genome Amplification Kit (Sigma-Aldrich, St. Louis, MO, USA; cat# WGA1). FISH was performed according to standard protocols for paraffin-embedded tissue sections and cryosections [21].

To unmask the target DNA, deparaffinized sections were treated with 100 μg/mL proteinase K in 25 mM Tris-Cl (pH 7.4) for 5 min at RT and then fixed in 4% PFA in PBS for 10 min at RT. After washing twice in PBS, sections were incubated in 50% formamide in 2xSSC at +4 °C overnight.

Cryosections were rehydrated in PBS for 15 min and then permeabilized in 0.5% Triton X-100 (Sigma-Aldrich, St. Louis, MO, USA, cat# T8787-100 ML) in PBS for 20 min. Unmasking the target DNA was performed in 10 mM sodium citrate buffer (pH 6.0) by a series of heating pulses in a microwave. After washing on 2xSSC slides were incubated in 50% formamide in 2xSSC at +4 °C overnight.

Both paraffin sections and cryosections with the mounted probe were incubated for 1 h at 37 °C. Cellular and probe DNA were denatured simultaneously for 5 min at 85 °C (for paraffin sections) or at 80 °C (for cryosections) and then hybridized for 1–2 days at 37 °C. Following post-hybridization washes, slides were mounted in Vectashield medium with DAPI (Vector Laboratories, Newark, CA, USA, cat# H-1200-10).

### 2.4. Microscopy

PAS-stained sections were captured using an Olympus BX63 microscope (equipped with an Olympus DP74 camera (both Olympus, Tokyo, Japan) and cellSENS Standard Microscope Imaging Software v.3.2 (Evident, Tokyo, Japan). FISH-stained paraffin sections were imaged using an Axioplan 2 microscope (Zeiss, Oberkochen, Germany) fitted with appropriate filter cubes (#49, #10, #15) and ISIS4 software v.5.4 (METASystems GmbH, Altlußheim, Germany). FISH-stained cryosections were imaged using the Olympus Fluoview FV3000 confocal laser scanning microscope and Olympus FV31S-SW viewer software v.2.6.1.243 (Olympus, Tokyo, Japan). To analyze confocal images of cryosections we used an image processing package Fiji (Fiji Is Just ImageJ v.2.17.0) [22]. Brightness and contrast of all images were adjusted using Corel PaintShop Photo Pro X6 v.16.1.0.48 (Alludo, Ottawa, ON, Canada).

For histological staging, sections were selected randomly from the cutting series. We analyzed every sixth section in the cutting series, systematically skipping the five intermediate sections to avoid sampling adjacent or overlapping regions of the same seminiferous tubule. A minimum of 100 fields of view were analyzed per bird.

Only seminiferous tubules with a Johnsen score = 10 (complete spermatogenesis and well-organized tubules) were included in the analysis.

### 2.5. Statistics

Histological data from the three birds were pooled for analysis, as the Kruskal–Wallis test indicated no significant differences among them (*p* ≥ 0.05). Dunn’s test with Holm correction for multiple comparisons was used to compare the relative numbers of each cell type at specific stages of the seminiferous epithelium cycle. All analyses were carried out in R (v4.4.2) [23].

## 3. Results

**Figure 1:**
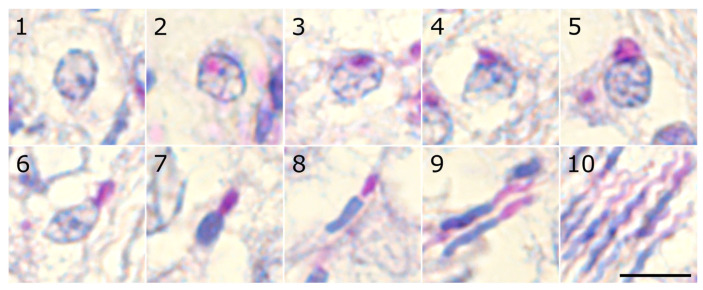
Micrographs of histological sections of zebra finch testes after PAS staining representing steps 1–10 of the spermiogenesis. Scale bar—5 μm.

To identify and characterize the stages of the seminiferous epithelium cycle in the zebra finch, we first categorized spermiogenesis in this species into ten distinct steps based on the progression of acrosomal development and nuclear morphology (Table 1, Figure 1). The first six steps represent round early spermatids, steps 7–8 represent intermediate spermatids, and steps 9–10 represent late spermatids.

**Figure 2:**
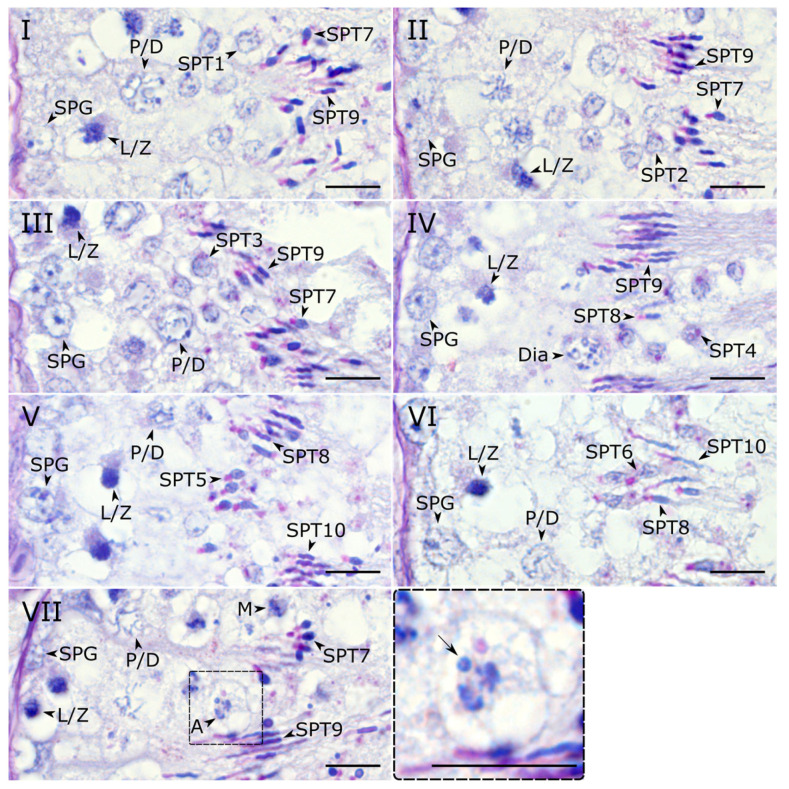
Microphotographs of histological sections of zebra finch testes after PAS staining representing stages (**I**–**VII**) of the seminiferous epithelium cycle. In all micrographs, the basal lamina of the seminiferous tubule is located at the left edge. SPG—spermatogonium; L/Z—leptotene/zygotene spermatocyte; P/D—pachytene/diplotene spermatocyte; Dia—diakinetic spermatocyte; M—metaphase spermatocyte; A—anaphase spermatocyte; SPT1-10, spermatid at step 1–10. The insert shows close-up of the anaphase cell with the micronucleus (arrow). Scale bar—10 μm.

Using the classification of spermiogenesis steps, we analyzed associations of spermatids at different steps and identified seven distinct stages of the seminiferous epithelium cycle (Figure 2). The seminiferous epithelium of the zebra finch exhibited a heterogeneous organization, which is typical for birds. Multiple stages of the seminiferous epithelium cycle were observed within a single cross-section (Supplementary Figure S1). We found spermatids from two (stage VII) or three (stages I–VI) different steps per stage. This is in contrast to the pattern described for mammals and the few non-passerine birds studied to date, where each stage typically contains spermatids at only one or two steps.

The Periodic acid–Schiff (PAS) staining visualizes acrosome development in spermatids. However, we could not distinguish secondary spermatocytes and step 1 spermatids, in which the acrosomal vesicle is not yet visible. Therefore, we merged these two cell types. Spermatogonia and both leptotene/zygotene and pachytene/diplotene spermatocytes were present throughout all stages of the cycle (Figure 2 and Figure S2, Supplementary Table S1).

**Stage I.** Early-round spermatids at step 1 are present (Table 2). The epithelium also contains intermediate, squid-like step 7 spermatids with oval acrosomes and highly elongated step 9 late spermatids exhibiting a characteristic double bend of the nucleus.

**Stage II.** Step 1 spermatids are replaced by step 2 spermatids. The acrosomes of the step 7 (squid-like) spermatids begin to extend, while the step 9 spermatids develop a more complex morphology with three or more bends. This stage is also characterized by the occasional presence of diakinetic spermatocytes.

**Stage III.** Spermatids at step 3 are present. This stage is also characterized by the transition from squid-like (step 7) to candle-like (step 8) intermediate spermatids. The late step 9 spermatids persist with multiple bends, and their acrosomes now show two curves. Diakinetic spermatocytes are sometimes observed.

**Stage IV.** Step 3 spermatids are replaced by step 4 spermatids. The intermediate spermatid population is predominantly composed of candle-like forms (step 8), with squid-like step 7 spermatids being rare. The morphology of the late step 9 spermatids remains consistent with stage III. At this stage, we occasionally observed the diakinetic spermatocytes.

**Stage V.** Spermatids at step 5 are present. The candle-like step 8 spermatids exhibit a single distinct bend. Step 9 spermatids develop into step 10 spermatids. Their acrosomes reach the maximum length and display more than three bends. A key meiotic transition occurs here: diakinetic spermatocytes are still occasionally observed, while metaphase spermatocytes appear.

**Stage VI.** Spermatids at step 6 are present. The morphology of the intermediate (step 8) and late (step 10) spermatids is similar to the previous stage. Both metaphase spermatocytes and anaphase spermatocytes are sometimes observed. This stage is the last one before spermiation.

**Stage VII.** Step 6 spermatids are matured to the intermediate step 7 spermatids. Their nuclei are transitioning from light to dark staining. The candle-like step 8 spermatids develop into the late step 9 spermatids. They have two bends and have initiated acrosomal elongation. Both metaphase and anaphase spermatocytes are occasionally present.

**Table 2:** Presence (+) and absence (-) of different germ cell types at stages I–VII of the seminiferous epithelium cycle in zebra finch.

Stage	SPG *	L/Z *	P/D *	Dia *	M *	A *	SPT *
I	+	+	+	-	-	-	1, 7, 9
II	+	+	+	-	-	-	2, 7, 9
III	+	+	+	+	-	-	3, 7/8, 9
IV	+	+	+	+	-	-	4, 8, 9
V	+	+	+	+	+	-	5, 8, 10
VI	+	+	+	-	+	+	6, 8, 10
VII	+	+	+	-	+	+	7, 9

* SPG—spermatogonia; L/Z—leptotene/zygotene spermatocytes; P/D—pachytene/diplotene spermatocytes; Dia—diakinetic spermatocytes; M—metaphase spermatocytes; A—anaphase spermatocytes; SPT—spermatids at steps 1–10.

**Figure 3:**
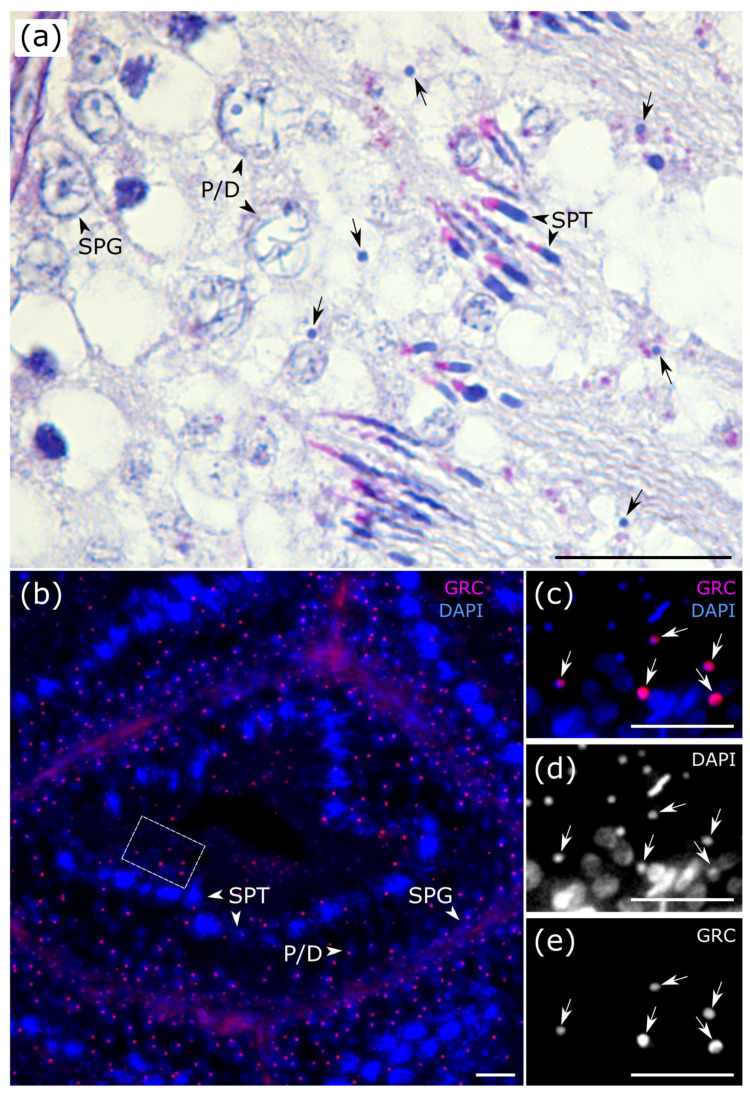
Visualization of GRC in testis sections of the zebra finch. Paraffin testis sections after PAS staining (**a**) and FISH (**b**) with GRC specific probe (red) and DAPI staining (blue). SPG—spermatogonium; P/D—pachytene/diplotene spermatocyte; SPT—spermatid. (**c**–**e**) Close-up shows GRC-positive micronuclei in the tubular lumen. Arrows point to micronuclei. Scale bar—20 μm.

At all stages of the seminiferous epithelium cycle, we observed micronuclei, which probably contained the eliminated GRC (Figure 3a). To visualize the GRC across all stages of spermatogenesis, we performed fluorescence in situ hybridization (FISH) using a GRC-specific probe. The GRC signal was detected in the nuclei of spermatogonia and primary spermatocytes located at the tubular periphery (Figure 3b and Figure S3). In contrast, GRC-positive micronuclei were predominantly localized within the tubular lumen (Figure 3b–e), indicating that the eliminated chromosome has been ejected from the spermatids. On a PAS-stained section, at stage VII, we observed an anaphase cell containing a distinct micronucleus (Figure 2VII). We suggest that this cell represents a secondary spermatocyte at anaphase II, with the micronucleus enclosing the eliminated GRC.

**Figure 4:**
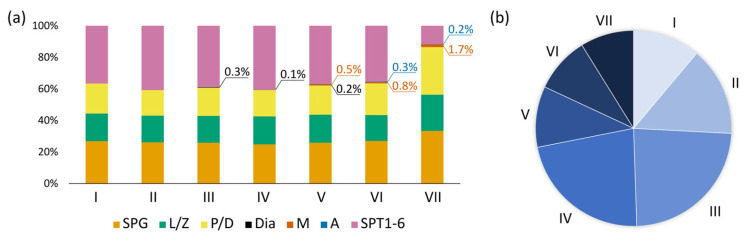
Relative distribution (**a**) of different germ cell types across stages I–VII of seminiferous epithelium cycle and the frequency (**b**) of these stages in zebra finch. SPG—spermatogonia; L/Z—leptotene/zygotene spermatocytes; P/D—pachytene/diplotene spermatocytes; Dia—diakinetic spermatocytes; M—metaphase spermatocytes; A—anaphase spermatocytes; SPT1-6—spermatids at steps 1–6. The percentage values indicate the proportion of minor germ cell types present at different stages.

We evaluated the distribution of various germ cell types across the different stages of the seminiferous epithelium cycle (Figure 4a, Supplementary Table S1). The relative proportions of germ cells showed little variation across most stages. However, two stages exhibited prominent differences. Stage III differed from almost all other stages by the higher relative number of diakinetic spermatocytes (Dunn’s, *p* < 0.05). Stage VII differed from other stages by the ratio of cell types. The relative number of spermatogonia, leptotene/zygotene spermatocytes, and pachytene/diplotene spermatocytes increased significantly (Dunn’s test, *p* < 0.05), whereas the proportion of early spermatids decreased (Dunn’s test, *p* < 0.05). We determined the frequency of occurrence of each stage of the cycle (Figure 4b). Stages III and IV were the most prevalent, whereas stages V–VII were the least common, which may indicate that the latter were shorter.

**Figure 5:**
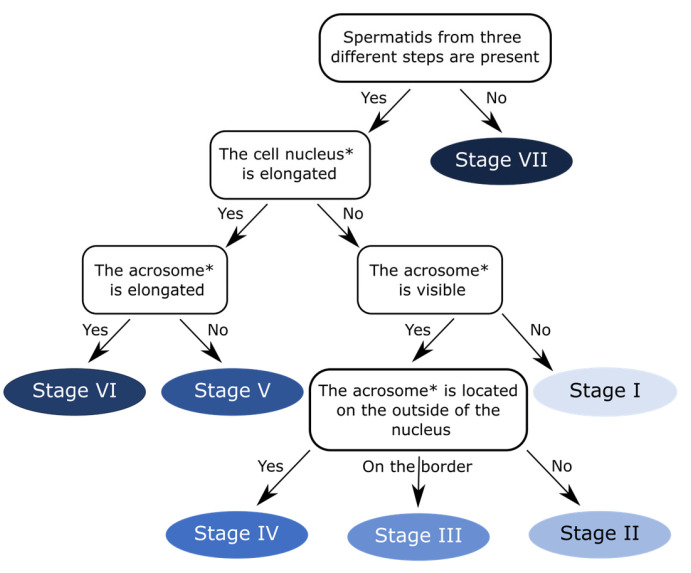
Flowchart for determining stages I–VII of seminiferous epithelial cycle in zebra finch. * Only the early spermatids at steps 1–6 should be analyzed.

To facilitate determination of seminiferous epithelial cycle stages in the zebra finch and other passerine birds, we created a user-friendly flowchart (Figure 5).

## 4. Discussion

The present study delineates ten distinct steps of spermiogenesis in the zebra finch and, for the first time, defines seven stages of the seminiferous epithelial cycle in the passerine birds. While previous studies in passerine birds have described only the steps of spermiogenesis [3,13,14], this comprehensive classification, based on acrosomal and nuclear morphological changes, reveals both similarities and unique characteristics of passerine spermatogenesis when compared to other avian species.

We observed multiple stages of the seminiferous epithelium cycle in a single tubular cross-section of zebra finch. This mosaic organization of the seminiferous epithelium was consistent with the pattern described for non-passerine birds [24,25].

However, we identified a difference in the number of stages and their cellular composition. While eight to ten stages are typically defined in non-passerine birds [1,9,24,26], we delineated only seven distinct stages in the zebra finch. This difference is likely determined by their unique spermatid associations. The zebra finch seminiferous epithelium contained spermatids at two or three developmental steps, in contrast to one or two steps typical of other birds and mammals. We hypothesize that this cellular organization, characterized by the presence of spermatids at an additional step per stage, may facilitate a faster progression of the entire seminiferous epithelial cycle compared to that in the non-passerine birds.

The number of spermiogenesis steps identified in the zebra finch (ten) is similar to that of the house sparrow (six steps divided into 10 substeps) [13], but differs from that of the masked weaver (12 steps) [3] and the carib grackle (14 steps) [14]. This variation is not uncommon. Within the Galloanseres clade, the number of spermiogenesis steps varies from 10 [1,5] to 12 [12,26,27]. It likely reflects species-specific differences in the dynamics and morphology of spermatid transformation or, more importantly, methodological differences in defining developmental stages. For example, in the guinea fowl (*Numida meleagris*), different authors have classified spermiogenesis into either 10 [24] or 11 steps [9].

Our detection of a micronucleus within an anaphase spermatocyte supports earlier cytological observation of GRC micronuclei within the cytoplasm of secondary spermatocytes [28]. Together, they indicate that the eliminated GRC is consistently packaged into a micronucleus that persists in the cytoplasm after meiosis. FISH analysis reveals the further fate of GRC micronuclei. The predominant localization of GRC-positive micronuclei within the tubular lumen suggests they are expelled from spermatids during their extensive cellular reorganization accompanied by cytoplasmic exclusion. This evidence supports a coherent model for GRC disposal: the chromosome is lagged and sequestered into a micronucleus during meiotic anaphase, which is then discarded from the developing spermatid prior to its final maturation.

Future studies using live-cell imaging are needed to confirm the mechanism of GRC elimination during male meiosis and to quantify the duration of the seminiferous epithelial cycle in the zebra finch.

## 5. Conclusions

In this study, we provide the first classification of the seminiferous epithelial cycle stages in the zebra finch, based on an analysis of ten distinct spermiogenesis steps. Our results demonstrate that the basic organization of the seminiferous epithelium in the zebra finch is consistent with that of other birds. However, the presence of spermatids at three different steps throughout most of the cycle highlights unique characteristics of spermatogenesis in passerines.

Our results show that the eliminated GRC persists as a micronucleus in the cytoplasm of secondary spermatocytes, confirming earlier cytological observations. FISH analysis further revealed that GRC-positive micronuclei are ejected into the tubular lumen, likely through cytoplasmic exclusion during spermatid remodeling.

This study provides an essential framework not only for reproductive biology but also for interpreting experimental manipulations in hormonal, genetic, and toxicological studies.

## Figures and Tables

**Table 1:** Steps of spermiogenesis in zebra finch.

Step	Description
1	Newly formed spermatids exhibit round nuclei with lightly stained nucleoplasm. Acrosomal vesicles are not yet visible.
2	A small acrosomal vesicle first appears in the cytoplasm of the cell.
3	The prominent acrosomal vesicle is attached to the nuclear membrane.
4	The acrosomal vesicle increases in size and begins to bulge away from the nucleus. The nuclear shape is generally spherical, though occasionally slightly ovoid or triangular.
5	Nuclear elongation commences, accompanied by increased chromatin condensation. The acrosomal vesicle further increases in size but retains a rounded shape.
6	Nuclear morphology remains similar to Step 5. The acrosomal vesicle begins to elongate.
7	Spermatids display elongated, squid-like, darkly stained nuclei. The acrosome increases in size and adopts an oval shape.
8	The nucleus elongates further, assuming a slender, candle-like morphology. The acrosome becomes pointed, narrower, and elongated, sometimes exhibiting a single bend.
9	Both the nucleus and acrosome continue to narrow, each developing 2–3 distinct bends.
10	The mature spermatids are prepared for spermiation. Both the elongated nucleus and acrosome reach their maximum length and exhibit multiple bends.

## Data Availability

The data presented in this study are available in the article and Supplementary Material.

## Supplementary Materials

The following supporting information can be downloaded at: https://www.mdpi.com/article/10.3390/ani15233427/s1, Table S1: Representation of the stages of the seminiferous epithelium cycle and cellular composition throughout the cycle; Figure S1: Microphotograph of the histological section of zebra finch testes after PAS staining. Roman numerals indicate the stages of the seminiferous epithelium cycle. The dotted lines demarcate the areas of the seminiferous tubule that are at different stages. SPG—spermatogonium; L/Z—leptotene/zygotene spermatocyte; P/D—pachytene/diplotene spermatocyte; SPT3-10, spermatid at step 3–10. Scale—20 μm; Figure S2: Micrographs of histological sections of zebra finch testes after PAS staining representing different germ cell types: (a) spermatogonium, (b) leptotene/zygotene spermatocyte, (c) pachytene/diplotene spermatocyte, (f) diakinetic spermatocyte, (e) metaphase spermatocyte, (f) anaphase spermatocyte. Scale—5 μm; Figure S3. Visualization of GRC in testis cryosections of the zebra finch. Cryosections after FISH (a) with GRC specific probe (green) and DAPI staining (blue). SPG—spermatogonium; P/D—pachytene/diplotene spermatocyte; SPT—spermatid. (b-d) Close-up shows GRC-positive micronuclei in the tubular lumen. Scale bar—20 μm.
