# Supplementary material for: Description of the Seminiferous Epithelium Cycle Stages in the Zebra Finch Taeniopygia guttata
Source: Animals (Basel). 2025 Nov 27;15(23):3427. doi: 10.3390/ani15233427 (PMC12691532; doi:10.3390/ani15233427)
Supplement: Supplementary file 1 [file animals-15-03427-s001.zip › Supplementary_Figures.pdf]

Supplementary Materials for:

Description of the seminiferous epithelium cycle stages in the Zebra Finch *Taeniopygia guttata*

Tatiana Bikchurina [1,2], Daria Rubtsova [2], Daria Odnoprienko [2], Pavel Borodin [2], Anna Torgasheva [3,4], and Lyubov Malinovskaya [1,2]*

[1] Laboratory of Genome Structure and Function; Novosibirsk State University, Novosibirsk, Russia
[2] Laboratory of Recombination and Segregation Analysis, Institute of Cytology and Genetics, Novosibirsk, Russia
[3] Centre for Molecular Biodiversity Research, Leibniz Institute for the Analysis of Biodiversity Change, Bonn, Germany
[4] Bonn Institute for Organismic Biology, University of Bonn, Bonn, Germany

This PDF file includes:

Figures S1 to S3

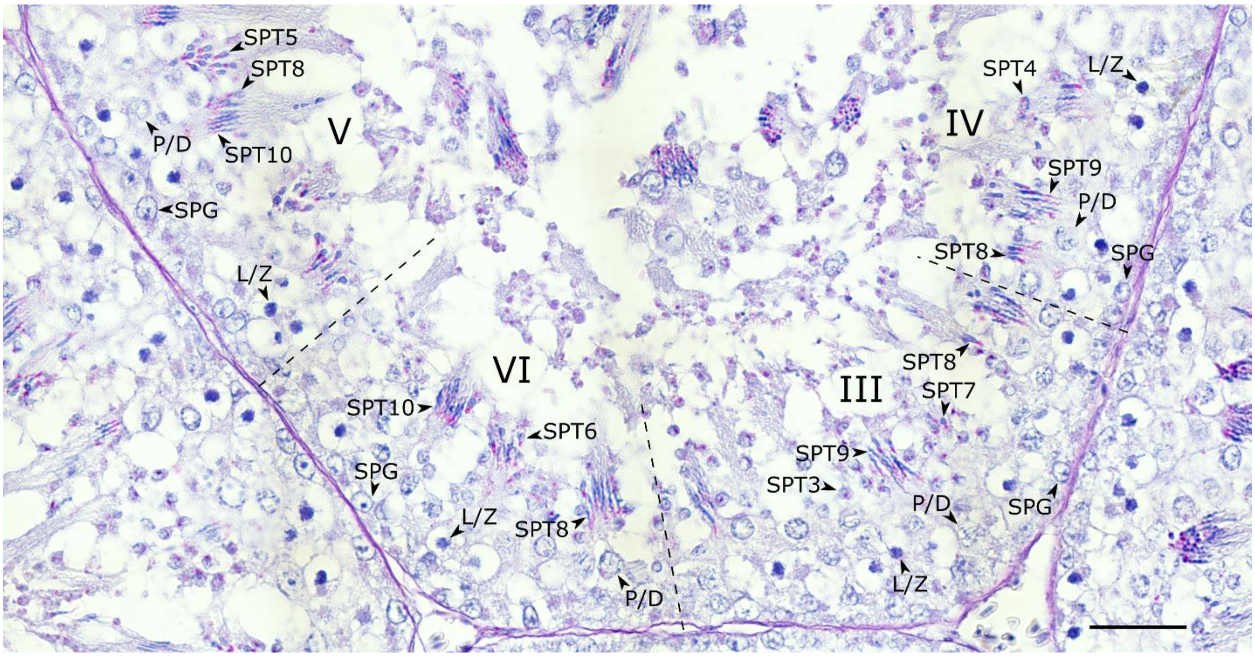

Figure S1. Microphotograph of the histological section of zebra finch testes after PAS staining. Roman numerals indicate the stages of the seminiferous epithelium cycle. The dotted lines demarcate the areas of the seminiferous tubule that are at different stages. SPG – spermatogonium; L/Z – leptotene/zygotene spermatocyte; P/D – pachytene/diplotene spermatocyte; SPT3-10, spermatid at step 3-10. Scale bar - 20 µm.

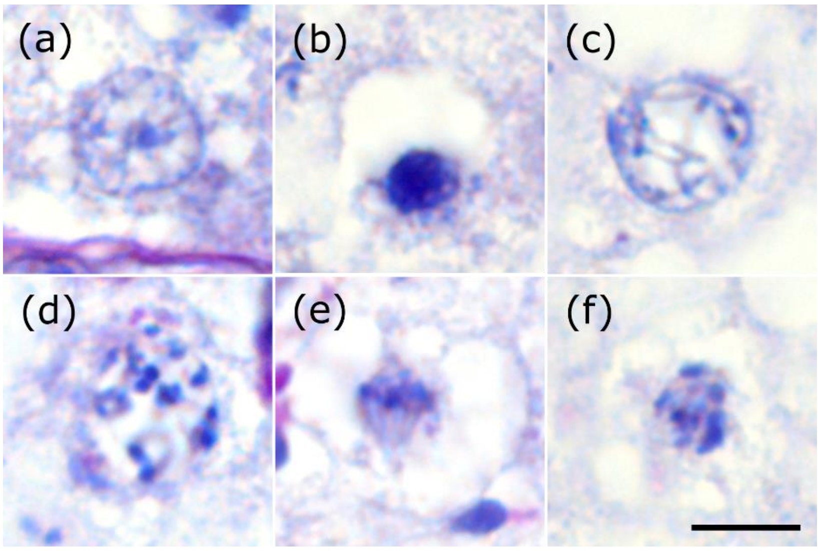

Figure S2. Micrographs of histological sections of zebra finch testes after PAS staining representing different germ cell types: (a) spermatogonium, (b) leptotene/zygotene spermatocyte, (c) pachytene/diplotene spermatocyte, (f) diakinetic spermatocyte, (e) metaphase spermatocyte, (f) anaphase spermatocyte. Scale bar - 5 μm.

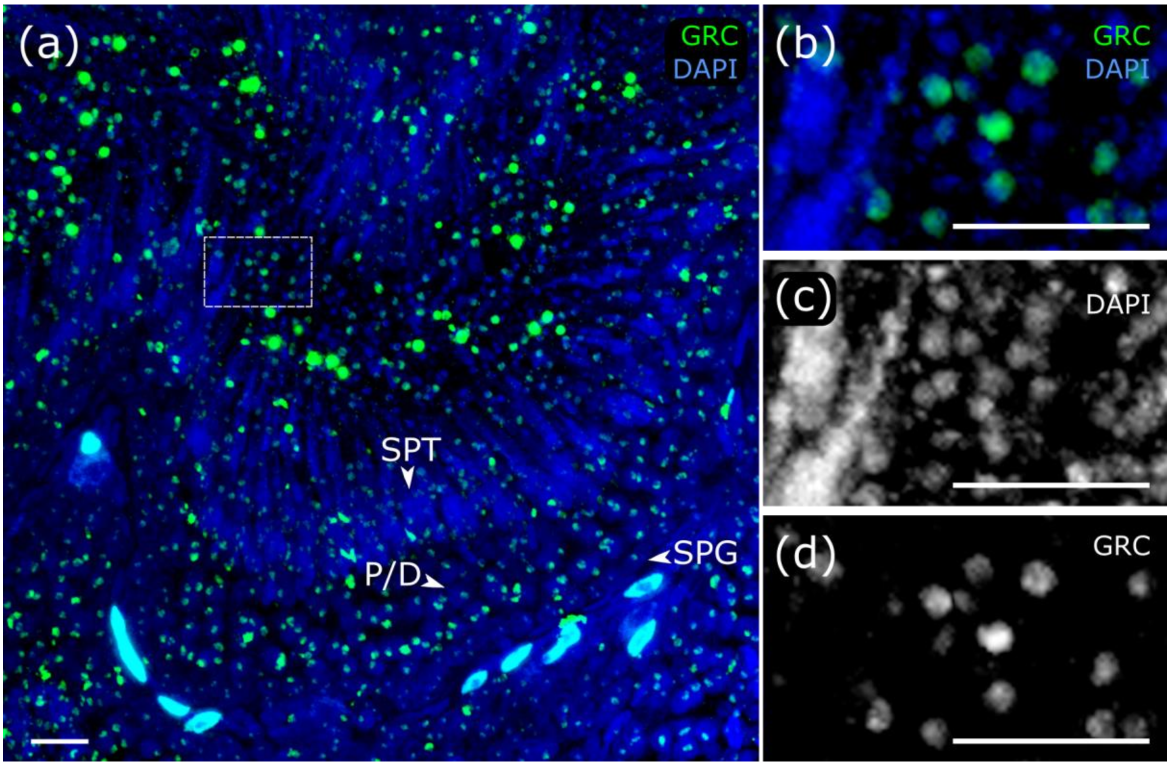

Figure S3. Visualization of GRC in testis cryosections of the zebra finch. Cryosections after FISH (a) with GRC specific probe (green) and DAPI staining (blue). SPG – spermatogonium; P/D – pachytene/diplotene spermatocyte; SPT – spermatid. (b-d) Close-up shows GRC-positive micronuclei in the tubular lumen. Scale bar – 20 μm.
